# Nonadherence to lipid‐lowering therapy and strategies to improve adherence in patients with atherosclerotic cardiovascular disease

**DOI:** 10.1002/clc.23935

**Published:** 2022-10-20

**Authors:** Nihar R. Desai, Michael Farbaniec, Dean G. Karalis

**Affiliations:** ^1^ Yale School of Medicine, Cardiovascular Medicine Section New Haven Connecticut USA; ^2^ Heart and Vascular Institute Penn State University Hershey Pennsylvania USA; ^3^ Department of Cardiology Thomas Jefferson University Philadelphia Pennsylvania USA

**Keywords:** adherence, atherosclerotic cardiovascular disease, inclisiran, lipid‐lowering, low‐density lipoprotein cholesterol

## Abstract

Despite the availability of effective therapies that lower low‐density lipoprotein cholesterol (LDL‐C) levels in patients with atherosclerotic cardiovascular disease, many eligible patients are inadequately treated and their LDL‐C levels remain suboptimal. Patient nonadherence to lipid‐lowering therapy (LLT) is a major contributor to the failure of LDL‐C goal attainment. Several factors have been identified as contributing to LLT nonadherence, including healthcare disparities due to socioeconomic status, age, race, sex, and cost; limited access to healthcare; perceived side effects associated with LLT; health literacy; and the presence of comorbidities. Suboptimal LLT use has also been associated with clinician factors, including failure to identify patients who require LDL‐C reassessment, insufficient LDL‐C monitoring, and clinical inertia such as a lack of therapy intensification. Several strategies to enhance LLT adherence have been shown to be effective, including the implementation of educational initiatives and tools for both patients and physicians, the use of clinical protocols and algorithms to identify patients at risk and optimize treatment, and improvements in electronic healthcare records. Pharmacy‐based programs designed to help patients with prescription refills, including reminders or the use of prescription delivery by mail, have also proven effective. Drugs requiring frequent administration can represent a barrier to treatment adherence; therefore, newer, more effective LLTs with lower frequency of administration and lower potential for polypharmacy may improve patient adherence to LLT. Implementation of strategies to identify patients at risk for LLT nonadherence and the use of flexible tools such as telemedicine to overcome geographical barriers may improve LLT adherence.

## 1 INTRODUCTION

Dyslipidemia is a major risk factor for developing atherosclerotic cardiovascular disease (ASCVD), a leading cause of cardiovascular (CV) morbidity and mortality in the United States.^1^ Recent cholesterol management guidelines targeting dyslipidemia have set progressively lower target threshold levels to achieve a reduction in the risk of ASCVD or associated clinical events.^2^, ^3^, ^4^, ^5^ The 2018 American College of Cardiology (ACC)/American Heart Association (AHA) guidelines on cholesterol management recommend using statins at maximally tolerated doses to achieve ≥ 50% lower low‐density lipoprotein cholesterol (LDL‐C) levels in patients with clinical ASCVD.^2^ Similarly, the National Lipid Association recommends an LDL‐C goal of <70 mg/dl for patients with ASCVD.^4^ In contrast, the 2019 European Society of Cardiology guidelines include a ≥ 50% LDL‐C reduction, and additional recommendation of an LDL‐C goal of <55 mg/dl for all patients with ASCVD.^3^

Despite the evolution to increasingly strict lipid guideline recommendations, many potentially treatable patients remain untreated, are inadequately treated, or are non‐adherent to medication, with the consequence of suboptimal LDL‐C levels.^6^ Indeed, patterns of poor lipid‐lowering therapy (LLT) implementation have been documented in observational studies, including the National Health and Nutrition Examination Survey 2011–2012, which found that up to 80% of patients with ASCVD who were statin‐eligible did not achieve their recommended LDL‐C goal of <70 mg/dl.^6^ Additionally, high rates of statin discontinuation have been reported in previous studies, including a cohort study of Medicare beneficiaries hospitalized for myocardial infarction (MI), showing that 15.4% of beneficiaries discontinued statins in the 6 months following hospital discharge.^7^

Multifactorial contributors to not achieving LDL‐C goals often include both patient and physician factors. One major contributor among eligible patients with ASCVD is patient nonadherence to LLT, with key drivers that include socioeconomic factors, limited access to healthcare, treatment side effects, and the presence of comorbidities.^8^, ^9^, ^10^ These factors may affect patient adherence to LLT at different timepoints, with cost, healthcare disparities, or access to healthcare being likely associated with early nonadherence, and treatment side effects and polypharmacy contributing to later nonadherence.

Adherence is critical for LLT effectiveness, with cumulative evidence linking poor LLT adherence and statin intolerance with worse outcomes and increased mortality.^11^ A retrospective cohort study of Medicare beneficiaries hospitalized for MI between January 1, 2007, and December 31, 2013, showed that statin intolerance was associated with a 36% higher rate of recurrent MI (41.1 vs. 30.1 per 1000 person‐years) and a 43% higher rate of coronary heart disease events (62.5 vs. 43.8 per 1000 person‐years) compared with patients with high statin adherence.^11^ Understanding the factors driving LLT nonadherence will help focus adherence improvement efforts to drive better patient outcomes in ASCVD.

Among physician behaviors, contributors to suboptimal patient LDL‐C levels include the under‐prescribing of guideline‐recommended LLT, a lack of treatment intensification, and clinical inertia (e.g., insufficient monitoring of LLT adherence and efficacy through lipid panels).^2^, ^9^

Herein we review factors reported to contribute to nonadherence to statin therapy and suboptimal LDL‐C levels in high‐risk patients prescribed statin therapy for ASCVD secondary prevention. Possible strategies for improving adherence to statins, and the use of new nonstatin LLT to increase treatment adherence will be discussed.

## 2 CHALLENGES FOR ADHERENCE TO CURRENTLY AVAILABLE LLTS

### 2.1 Patient‐related factors

#### 2.1.1 Poor ASCVD awareness and unintentional nonadherence

A poor understanding of ASCVD and associated health risks and a limited appreciation of the benefits of treatment to reduce hypercholesterolemia represent another barrier to LLT adherence. This barrier is in part a consequence of physicians overestimating patients' understanding of the importance of managing CV risks.^12^

In addition, poor LLT adherence can sometimes be unintentional and a consequence of forgetfulness or a lack of understanding of medication instructions. A cross‐sectional questionnaire study of stroke survivors showed no association between nonadherence to preventive drugs and personal beliefs about medicine and suggested that nonadherence might be associated with difficulties with memory and the need of help from next of kin.^13^

### 2.2 Therapy‐related factors

#### 2.2.1 Side effects associated with LLT

**Figure 1:**
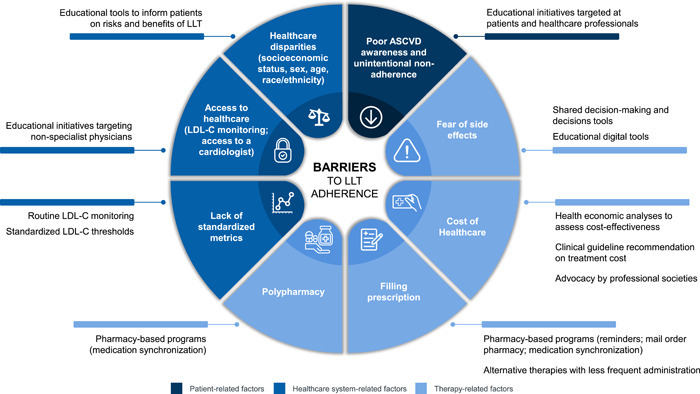
Barriers to adherence to LLT and strategies for improvement. LDL‐C, low‐density lipoprotein cholesterol; LLT, lipid‐lowering therapy.

Common reasons for declining or discontinuing LLT use include a fear of actual or perceived side effects.^9^ Although side effects with LLT are uncommon, statins may cause muscle symptoms and increase the risk of type 2 diabetes, use of ezetimibe may cause gastrointestinal side effects and muscle symptoms, and antiproprotein convertase subtilisin/kexin type 9 (PCSK9) monoclonal antibodies (mAbs) may cause a local site reaction, influenza‐like symptoms, and nasopharyngitis.^9^, ^14^, ^15^, ^16^, ^17^ In an analysis of data from the Patient and Provider Assessment of Lipid Management (PALM) registry, among 5693 adults recommended for statin therapy, 1511 (26.5%) were not on statin therapy, including 153 patients who declined statin treatment and 464 who discontinued treatment.^9^ Among untreated patients, fear of side effects (36.8% of patients) was the most common reason cited for declining statin treatment, whereas perceived side effects (55.0% of patients) was the most frequent reason reported for statin discontinuation (Figure 1; Table 1).^9^ Severe adverse effects associated with statins, such as increased risk of myopathy and rhabdomyolysis, are rare. However, many patients discontinue statin treatment because they believe that statins frequently cause muscle pain, which has been supported by observational studies.^15^ Recent double‐blinded n‐of‐1 trials in patients with severe muscle symptoms during statin treatment showed no significant effects of statins on muscle pain compared with placebo, with a low number of patients withdrawing from the trials. This finding suggests that muscle symptoms may be associated with a nocebo effect among statin users.^18^

**Table 1:** Key studies identifying barriers to adherence to LLT

Barrier	Study	Study design	Key findings
Healthcare system‐related barriers			
Healthcare disparities	Bradley et al.^9^	PALM registry; enrollment May 2015 to November 2015 Analysis: descriptive statistics and multivariable model with Poisson regression Limitation: patient self‐reported statin use	47.0% of untreated, statin‐eligible patients were never offered a statin Compared with current statin users, patients who were never offered a statin were more likely to be female (51.1% vs. 39.4%) and of Black (20.9% vs. 12.1%) or Hispanic (14.0% vs. 10.1%) ethnicity Increased likelihood of never being offered statin therapy for Black versus White patients, having no health insurance versus having private health insurance, and female versus male sex
	Ingersgaard et al.^10^	Systematic review of reviews Limitation: suboptimal quality of the included reviews	Factors associated with positive impact on adherence: high socioeconomic and educational levels and middle age Factor associated with negative impact on adherence: female sex, older/younger age, nonwhite race, low socioeconomic level, high copayments, first‐time statin use, and comorbidities
	Suero‐Abreu et al.^19^	Retrospective analysis of patient visits	Black race was independently associated with statin under‐treatment (OR: 0.42; 95% CI: 0.23‒0.77; *p* = .005) Hispanic patients were more likely than Black patients to be appropriately treated with statin therapy (86.8% vs. 77.2%)
Access to healthcare	Jia et al.^20^	Database study of the Veterans Affairs healthcare system Analysis: adjusted regression models Limitations: only completed lipid panels were included	Significant association between routine completion of lipid panels and increase in statin adherence in patients with ASCVD on statins
	Nanna et al.^21^	PALM registry; enrollment May 2015 to November 2015 Analysis: multivariable generalized linear mixed modeling (adjusted data)	Significant variation in statin treatment among US clinics contributes to differences in patient LDL‐C levels
Therapy‐related barriers			
Fear of side effects	Bradley et al.^9^	Cross‐sectional registry	26.5% of adults recommended for statin treatment were not on a statin 36.8% of patients declined statin treatment due to fear of side effects 55.0% of patients discontinued statin treatment due to perceived side effects
Cost of healthcare	Khera et al.^8^	NHIS data from 2013 to 2017 Analysis: descriptive statistics and multivariate logistic regression model (adjusted data) Limitation: self‐reported ASCVD	12.6% of respondents reported cost‐related non‐adherence Nonadherence due to medication cost was a major challenge for patients who were <65 years of age, female, from low‐income families, or uninsured
	Myers et al.^22^	Healthcare claims study	61% anti‐PCSK9 mAb prescription rejection rate 15% prescription abandonment rate Higher risk of CV events among patients with anti‐PCSK9 mAb prescription denials (HR for composite CV event: 1.10; 95% CI: 1.01‒1.19; *p* = .02) or abandonment (HR for composite CV: 1.12; 95% CI: 1.01‒1.24; *p* = .03) compared with those with prescription approvals
Filling prescription	Qato et al.^23^	Pharmacy claims study at retail pharmacies that subsequently closed Analysis: multivariable linear regression models (adjusted data)	Pharmacy closures led to a decrease in adherence to statins and other CV medications

Abbreviations: ASCVD, atherosclerotic cardiovascular disease; CI, confidence interval; CV, cardiovascular; HCP, healthcare provider; HR, hazard ratio; LDL‐C, low‐density lipoprotein cholesterol; LLT, lipid‐lowering therapy; mAb, monoclonal antibody; NHIS, National Health Interview Survey; PALM, Patient and Provider Assessment of Lipid Management; OR, odds ratio; PCSK9, proprotein convertase subtilisin/kexin type 9.

#### 2.2.2 Access to medications

##### Cost

Financial toxicity, which refers to the growing economic burden associated with healthcare costs and their indirect consequences, affects millions of patients with ASCVD in the United States and is particularly prevalent among low‐income families, the uninsured, and those not eligible for Medicare.^24^ Treatment nonadherence was identified as a major consequence of health‐related costs among United States adults with a history of ASCVD, with 12.6% of respondents reporting cost‐related nonadherence, including skipping doses, taking lower than prescribed doses, and intentionally delaying prescriptions refills to defer costs.^8^ Furthermore, analyses conducted using a multivariate logistic regression model that accounted for confounding factors showed that nonadherence to LLT due to medication cost was a major challenge, particularly among individuals who are <65 years of age, are female, come from low‐income families, or are uninsured.^8^

Prescription rejections by payers are another consequence of high medication costs contributing to poor adherence and negative CV outcomes in ASCVD. While statins are available to United States patients as generics at low or no cost, other LLTs such as anti‐PCSK9 mAbs are costly, resulting in budget constraints for payers and financial implications for patients.^22^ A study of healthcare claims showed that anti‐PCSK9 mAb prescriptions had a 61% rejection rate and a 15% prescription abandonment rate, with a significantly higher risk of CV events among patients with anti‐PCSK9 mAb prescription denials (hazard ratio [HR] for composite CV event: 1.10; 95% CI: 1.01‒1.19; *p* = .02) or abandonment (HR for composite CV: 1.12; 95% CI: 1.01‒1.24; *p* = .03) compared with those with prescription approvals (Table 1).^22^

##### Inability to easily fill prescriptions

The inability to easily fill prescriptions represents another barrier to statin adherence, as demonstrated by a study of insurance claims from patients who filled prescriptions between January 1, 2011, and December 31, 2016, at retail pharmacies that subsequently closed.^23^ Among older adults, pharmacy closures resulted in a rapid decline in adherence to statins and other CV medications during the 3 months following closure, with the decline in prescription refills persisting over 12 months (Table 1).^23^ In a retrospective cohort study that used pharmacy claims data to identify patients initiating anti‐PCSK9 mAbs between January 1, 2016, and June 30, 2016, a large proportion of patients discontinued anti‐PCSK9 mAb therapy around the time frame for which their health plan would require recertification to continue to access the therapy.^25^

#### 2.2.3 Polypharmacy

Patients with ASCVD often present with numerous comorbidities, which inadvertently lead to polypharmacy, resulting in a requirement for careful treatment coordination and the potential for concurrent medications becoming a barrier to LLT adherence. Dyslipidemia is often present in patients with diabetes, hypertension, and/or obesity, and multifactorial interventions are required with these patients, including lifestyle modifications (diet, weight loss, and increased physical activity) and appropriate pharmacologic treatment, potentially including multiple medications. Polypharmacy is also often required to address residual CV risk in patients treated with long‐term LLT, potentially presenting an additional challenge to LLT adherence (Figure 1).^26^

In addition, the possibility of drug‐drug interactions of statins with other prescribed drugs increases with the number of concurrent medications, and these interactions may result in increased statin‐associated adverse effects.^27^

### 2.3 Healthcare system‐related factors

#### 2.3.1 Healthcare disparities

Healthcare disparities have been consistently reported in patterns of statin prescription and use, with differences based on race/ethnicity, sex, age, socioeconomic status, and co‐occurring vulnerabilities.^10^, ^19^, ^22^ Data from the PALM registry showed that among patients recommended for statin therapy for secondary prevention but not being treated (*n* = 566), 47.0% were never offered a statin by their provider.^9^ Compared with patients on statin therapy, those who were never offered a statin were more likely to be female (51.1% vs. 39.4%; *p* < .001) and of Black (20.9% vs. 12.1%) or Hispanic (14.0% vs. 10.1%; *p* = .0005) ethnicity.^9^ Furthermore, patients who were never offered a statin had lower rates of private insurance and college education, and lower household incomes.^9^ These findings were supported by multivariable analyses showing a decreased likelihood of ever being offered statin therapy for Black versus White patients, patients without health insurance versus with private health insurance, and female versus male patients. In a 2019 analysis of published systematic review studies that was conducted to identify factors affecting adherence to statins, high socioeconomic and educational levels as well as middle age (50−65 years) were identified as factors that positively influenced adherence.^10^ Conversely, female sex, older (>70 years), or younger (<50 years) age, nonwhite race, low socioeconomic level, high medication copayments, first‐time statin use, and comorbidities all negatively impacted adherence.^10^ Similarly, a retrospective study conducted in a large urban medical center in an underserved population composed of mainly Black (47.2%) and Hispanic (45.7%) patients showed that Black race was a factor independently associated with statin undertreatment (odds ratio [OR]: 0.42; 95% CI: 0.23‒0.77; *p* = .005).^19^ In the same study, Hispanic patients were found to be more likely than Black patients to be appropriately treated with statin therapy (86.8% vs. 77.2%) (Figure 1; Table 1).^19^ Additional research is needed to identify the causes underlying these healthcare disparities and explain the lower adherence to statins observed among female and Black patients. Initial studies suggest that higher rates of statin intolerance and a lower awareness of CV disease (CVD) risk in women may play a role in the observed sex‐based disparities in statin adherence.^28^ Similarly, the higher rate of statin nonadherence observed among Black patients has been associated with increased medication and out‐of‐pocket costs.^29^

#### 2.3.2 Access to healthcare

Access to healthcare and associated treatment support is another established factor impacting LLT adherence.^20^, ^30^ One example of this association comes from a Veterans Affairs healthcare system study of patients with ASCVD on statin therapy who had at least one primary care visit between October 2013 and September 2014 that linked lipid monitoring frequency with adherence to statins, suggesting that lipid monitoring may improve statin adherence.^20^

The type of clinical practice visited may also impact compliance with statin guideline recommendations and treatment benefit. An analysis conducted in 74 United States clinics within the PALM registry showed that relative to clinical practices with lower statin use (lowest or middle tertile), those with higher statin use (highest tertile) were more likely to be cardiology practices (68.0% vs. 48.0% vs. 12.5%; *p* < .001), and that patients at highest tertile clinics were more likely to achieve LDL‐C < 70 mg/dl (adjusted OR: 1.49; 95% CI: 1.08‒2.04) or <100 mg/dl (adjusted OR: 1.78; 95% CI: 1.41‒2.25) compared with patients at the lowest tertile clinics.^21^ The study reported that adoption of the 2013 ACC/AHA cholesterol guidelines was higher among physicians in the highest tertile practices (80.2%) compared with those in the mid‐ or lowest tertile practices (67.8% vs. 59.3%; *p* = .003). Moreover, physicians in the highest tertile practices were more likely to believe in statin safety (72.8% vs. 69.8% vs. 56.6%; *p* < .05) and efficacy (79.0% vs. 72.1% vs. 53.7%; *p* < .001) than those in the lowest or middle tertile (Table 1).^21^ These data highlight a gap in the quality of CV care provided by cardiology practices versus primary care, family medicine, and internal medicine practices, and suggest that educational initiatives targeted at nonspecialist physicians could improve adherence to LLT.

#### 2.3.3 Lack of standardized metrics for LLT adherence

Variation between guidelines in their recommended monitoring metrics and thresholds represents another potential barrier to patient adherence to LLT. Evidence supporting the importance of adequate LDL‐C monitoring and the need for standardization comes from a Canadian study showing that, among patients who received percutaneous coronary interventions, only 52% had an LDL‐C measurement within 6 months.^31^ During 3 years of follow‐up, LDL‐C levels and CV events were positively correlated, suggesting that regular LDL‐C assessment and treatment optimization could lower CV event risk.^31^ Standardization of treatment metrics and routine LDL‐C monitoring across healthcare systems based on current guidelines will increase adherence and improve patient health.

## 3 EXISTING STRATEGIES AND TOOLS TO IMPROVE ADHERENCE TO CURRENT LLT

Various implementation strategies have been used to improve LLT‐use and clinical outcomes associated with hyperlipidemia. A systematic review and meta‐analysis conducted to determine which implementation strategies are more successful identified 258 strategies across 86 trials.^32^ Among them, data from 31 trials reporting outcomes of interest showed that implementation strategies aimed at improving statin use were effective in lowering LDL‐C (OR: 1.33; 95% CI: 1.13‒1.58; *p* = .0008), increasing rates of statin prescribing (OR: 2.21; 95% CI: 1.60‒3.06; *p* < .0001), and improving adherence (OR: 1.30; 95% CI: 1.04‒1.63; *p* = .023). The identified strategies were organized into the following categories:

1. Use evaluative and iterative strategies.
2. Support clinicians (i.e., create new clinical teams; facilitate relay of clinical data to providers; remind clinicians; revise professional roles).
3. Adapt and tailor to the context.
4. Engage consumers.
5. Train and educate the stakeholders.
6. Change infrastructure (i.e., mandate change; change record systems; create or change credentialing and/or licensure standards; change physical structure and equipment).
7. Develop stakeholder relationships.
8. Use financial strategies.
9. Provide interactive assistance.

No single strategy or category resulted in a greater impact; however, the concurrent use of multiple implementation strategies was associated with a greater reduction in LDL‐C (standardized mean difference: −0.38; 95% CI: −0.67 to −0.09; *p* = .05).^32^

### 3.1 Shared decision‐making and decision tools

Patient education and patient–physician communication, together with the use of adequate educational tools, are important to enhance the understanding of risk associated with ASCVD and willingness to initiate and adhere to LLT. A survey of patients in the PALM registry showed a significantly higher perception of ASCVD‐associated risk and treatment willingness when patients were shown lifetime ASCVD risk versus 10‐year ASCVD risk or CVD death risk.^33^ Furthermore, a significantly lower perception of risk and treatment willingness was observed when patients were shown pictograms versus bar graphs.^33^ These findings suggest that the process of shared decision‐making, wherein patients are engaged in making decisions on their own treatment, may improve adherence to LLT. Design and use of adequate decision aids that effectively communicate the risks and benefits associated with LLT may substantially impact treatment decisions.

### 3.2 USE of digital tools

Medical misinformation that may be widely spread through the internet and social media can contribute to patient unwillingness to initiate appropriate treatment, resulting in poor clinical outcomes.^34^ The development and appropriate use of digital tools is an important strategy to educate patients on the benefit of optimal LLT. In fact, digital tools have been developed by healthcare providers and are available for both patients and clinicians.^35^ For example, the Million Hearts initiative co‐led by the Centers for Disease Control and Prevention and the Centers for Medicare & Medicaid Services offers several online tools and resources for the management of dyslipidemia, including infographics, ASCVD risk estimators, diagnostic applications, social media educational messages, Q&A tools, and short videos.^35^

### 3.3 Pharmacy‐based programs

Pharmacy‐based programs and initiatives have also had a positive impact on LLT adherence, as demonstrated by a systematic review and meta‐analysis that compared initiative results from a pharmacist‐led intervention group versus a control group.^36^ Significantly higher adherence was observed in the intervention group (OR: 1.67; 95% CI: 1.38‒2.02; *p* < .001).^36^ Several studies have investigated pharmacist‐led initiatives designed to support patients with their prescription management. A population‐based study showed that automated telephone reminders to patients with CVD (diabetes, ASCVD, heart failure, and chronic kidney disease) who were nonadherent to statins resulted in a significant increase in the proportion of patients who refilled their statin prescription within 2 weeks (30.3% vs. 24.9%, *p* < .0001).^37^ A database study of patients discharged with ischemic stroke compared medication adherence between patients who used a mail order pharmacy versus a local pharmacy.^38^ Adherence was assessed using the continuous medication gap (CMG) score, which represents the proportion of days for which no medication was available to the patient. Higher adherence to treatment was observed in patients using a mail order pharmacy versus a local pharmacy (CMG adherence: local pharmacy group, 0.28; mail order pharmacy group, 0.11; *p* < .001), suggesting that easy access to medications through a mail order pharmacy may also improve treatment adherence in other disease states such as ASCVD.^38^ Further support comes from an analysis of patients participating in Time My Meds, an appointment‐based medication synchronization program in community pharmacies, which showed higher patient adherence to statins (97.6% vs. 75.0%) compared with patients receiving usual care.^39^

Patient management of multiple medication refills can also represent a challenge to statin treatment adherence. A retrospective claims analysis of Medicare Advantage patients taking multiple maintenance medications (i.e., antihypertensives, lipid‐lowering drugs, antidiabetic agents) compared patients with synchronized refill schedules versus patients without synchronized schedules.^40^ Patients in the synchronized refill group had better adherence to LLT than those in the control group, as measured by proportion of days covered (absolute difference in mean proportion of days covered score: 0.06; 95% CI: 0.06‒0.07; *p* < .01), and the proportion of LLT‐adherent patients was 15% higher in the synchronized refill group versus the control group (*p* < .01) (Figure 1).^40^ By contrast, another study among US military veterans at risk of CVD (LDL‐C > 130 mg/dl and/or <80% refill adherence of LLT in the last 12 months) tested the use of a special medication blister packaging labeled for daily use with a written reminder.^41^ The study did not find a significant difference in refill rates between the packaging intervention arm and the education‐only arm (mean difference: 7.6%; 95% CI: −5% to 20%; *p* ≤ .24).^41^

#### 3.3.1 Treatment frequency

For some patients, the frequency of drug administration could represent a barrier to LLT use, and therapies that require less frequent administration could help address adherence issues associated with frequent administration. For example, among potential LLTs, anti‐PCSK9 mAbs (e.g., alirocumab and evolocumab) that are administered every 2 weeks or monthly are effective at reducing LDL‐C levels by 45%−60%.^42^ Inclisiran is a first‐in‐class LDL‐C–lowering small interfering RNA that mimics the body's process of RNA interference to degrade PCSK9 mRNA and prevents the production of PCSK9 protein. Inclisiran's twice‐yearly subcutaneous dosing regimen by a healthcare professional results in an effective and sustained reduction in circulating LDL‐C levels of up to 52% from baseline.^43^ However, currently there are no published studies that directly assessed treatment adherence to anti‐PCSK9 mAbs or inclisiran, thus any benefit associated with their reduced administration frequency remains to be demonstrated. The availability of options for highly effective treatment of elevated LDL‐C with different administration requirements should broaden patient choice, aid adherence to LLT, and offer more cost‐effective options (Figure 1).

#### 3.3.2 The future of lipid management

It is critical that additional strategies be implemented to improve lipid management, including personalized treatment and development of tools to help identify patients who are nonadherent or who need to be reassessed, as well as strategies to increase the frequency of monitoring lipid levels (e.g., at‐home testing) to allow better medication adjustments and reduce exposure to elevated LDL‐C.^44^ Furthermore, the use of telemedicine could help overcome barriers posed by geographical distances to deliver continuous care directly to patients. Further studies are required to better understand the effectiveness of telemedicine in lipid management.^45^

Additional changes that could improve statin adherence and ASCVD outcomes include interventions to address the financial toxicity associated with ASCVD. One approach could be to assess the cost‐effectiveness of available LLTs using validated health economic models based on data from randomized clinical trials or postregistrational studies. Cost‐effectiveness rather than cost should be used to determine patient access to therapy.^46^ The active engagement of professional societies to address these challenges and to advocate for patient access to therapy should also benefit patients (Figure 1).

## 4 CONCLUSIONS

Despite the availability of effective therapies, a large gap exists between guideline‐recommended LLT and clinical practice, with a high percentage of eligible patients not receiving appropriate treatment and presenting with LDL‐C levels higher than their goal. Closing this gap would be the first step toward improving clinical outcomes in patients with ASCVD. Another major barrier to achieving LDL‐C goals is poor patient adherence to LLT due to multiple contributors including out‐of‐pocket costs, comorbidities, socioeconomic status, side effects, lack of access to healthcare, failure to identify eligible patients, and physician under prescribing and underdosing. There is an inverse correlation between long‐term statin adherence and mortality in patients with ASCVD, suggesting that improving adherence would lead to a significant increase in survival among these patients.

There is a need to implement new strategies to increase the use of LLT in eligible patients and to improve treatment adherence. Initiatives that have shown to be effective in increasing the use of LLT include patient and physician educational programs and algorithms to guide decision‐making, as well as pharmacy‐based programs and routine monitoring. Importantly, the ability of individual patients to access the most effective therapy, including use of non‐statin therapies that require less frequent administration, could help them to reach and maintain guideline‐recommended LDL‐C goals and overcome treatment non‐adherence.

## CONFLICTS OF INTEREST

Dean G. Karalis has received consulting fees from Amarin, Novartis, and Esperion. He is on the speakers bureau for Amarin and Esperion, and has received research grants from Amgen, Sanofi‐Regeneron, and Novartis. Nihar R. Desai works under contract with the Centers for Medicare and Medicaid Services to develop and maintain performance measures used for public reporting and pay for performance programs. He received research grants and/or consulting for Amgen, AstraZeneca, Boehringer Ingelheim, Bristol Myers Squibb, Cytokinetics, Novartis, scPharmaceuticals, and Vifor Pharma. The remaining author declares no conflict of interest

## Data Availability

Data sharing is not applicable to this article as no new data were created or analyzed in this study.
